# Surgical safety checklist compliance process as a moral hazard: An institutional ethnography

**DOI:** 10.1371/journal.pone.0298224

**Published:** 2024-02-26

**Authors:** Marcia Facey, Nancy Baxter, Melanie Hammond Mobilio, Elizabeth Peter, Carol-anne Moulton, Elise Paradis

**Affiliations:** 1 Leslie Dan Faculty of Pharmacy, University of Toronto, Toronto, Ontario, Canada; 2 Li Ka Shing Knowledge Institute, St Michael’s Hospital, Unity Health Toronto, Toronto, Ontario, Canada; 3 Melbourne School of Population and Global Health, The University of Melbourne, Melbourne, Victoria, Australia; 4 The Wilson Centre, University Health Network, Toronto, Ontario, Canada; 5 Institute of Health Policy, Management, and Evaluation, University of Toronto, Toronto, Ontario, Canada; 6 Lawrence S. Bloomberg Faculty of Nursing, University of Toronto, Toronto, Ontario, Canada; 7 Joint Centre for Bioethics, University of Toronto, Toronto, Ontario, Canada; 8 Department of Surgery, University of Toronto, Toronto, Ontario, Canada; Istinye University: Istinye Universitesi, TURKEY

## Abstract

**Background:**

Charting is an essential component of professional nursing practice and is arguably a key element of patient safety in surgery: without proper, objective, and timely documentation, both benign and tragical errors can occur. From surgery on wrong patients to wrong limbs, to the omission of antibiotics administration, many harms can happen in the operating room. Documentation has thus served as a safeguard for patient safety, professional responsibility, and professional accountability. In this context, we were puzzled by the practices we observed with respect to charting compliance with the surgical safety checklist (SSC) during a study of surgical teams in a large, urban teaching hospital in Canada (pseudonym ‘C&C’).

**Methods:**

This article leverages institutional ethnography and a subset of data from a larger study to describe and explain the social organisation of the system that monitored surgical safety compliance at C&C from the standpoint of operating room nurses. This data included fieldnotes from observations of 51 surgical cases, on-the-spot interviews with nurses, formal interviews with individuals who were involved in the design and implementation of the SSC, and open-ended questions from two rounds of survey of OR teams.

**Findings:**

We found that the compliance form and not the SSC itself formed the basis for reporting. To meet hospital accuracy in charting goals and legislated compliance documentation reporting requirements nurses ‘pre-charted’ compliance with the surgical checklist. The adoption of this workaround technically violated nursing charting principles and put them in ethically untenable positions.

**Conclusions:**

Documenting compliance of the SSC constituted a moral hazard, constrained nurses’ autonomy and moral agency, and obscured poor checklist adherence. The findings highlight how local and extra local texts, technologies and relations create ethical issues, raise questions about the effectiveness of resulting data for decision-making and contribute to ongoing conversations about nursing workarounds.

## Introduction

The surgical safety checklist (SSC) is a part of the patient safety and governance initiative that has been mandated in numerous jurisdictions. In Ontario, Canada it was accompanied by mandatory public reporting of safety indicators for compliance [1,2] with nurses being made responsible for documenting this compliance. ‘Compliance’ as part of the patient safety movement has been variously conceptualised and studied in numerous disciplines including medicine and nursing [3,4]. For the most part, it has been treated as a rational, cognitive and process that can be easily and objectively measured, as opposed to a highly complex activity that is informed by—and needs to be considered and understood within—its cultural and practice contexts [3–6].

In their dual roles as care providers and ‘guardians of patient safety’ [7], and per their professional standards and codes of ethics, nurses are obliged to keep accurate records. Charting is a key component of ethical and professional nursing practice and patient record keeping must be objective, accurate, and timely [8]. Charting is also a communication and collaboration tool that has been closely aligned with quality of care and patient safety [9]. Nursing charting practices have been the subject of study [e.g., 9–14], with many noting problems of incomplete, untimely, and inaccurate documentation practices as well as barriers to and suggestions for more effective protocols.

Researchers have suggested that there are similar inconsistencies with SSC documentation practices, with discrepancies between what is documented and what happens in reality [15,16]. Beyond stating that SSC documentation is often inaccurate or incomplete, or noting the disconnect between reported compliance rates and what is actually observed [e.g., 15,17], little detailed attention has been paid to nurses’ experience of the SSC documentation process in the context of the cultural practices of surgery. Using an institutional ethnographic approach (IE) [18–20], we trace nurses’ charting work—activities, knowledge and concerns—at a large urban hospital (pseudonym C&C) to answer the question, how can we understand and explain the disconnections in nurses’ surgical safety checklist charting practices?

## Methodology

An IE approach, with its requirement for detailed descriptions, offers a way of understanding and explicating how nurses’ work of charting SSC compliance is produced. Institutional ethnography rejects concepts such as *social structures* as explanations for why social world operates as it does; it posits instead that it is *accomplished* through human activity [21]. IE starts with a tension or a puzzle that is latent in, but arises from, everyday happenings. It is concerned with describing people’s everyday experiences related to these tensions or puzzles and explicating how these are organised and coordinated by institutional forces. ‘Institution’ in IE refers to complex arrangements or regimes expressed in texts such as policies, directives, legislations, discourses, technologies (e.g., electronic medical records (EMRs)), culture, training, and regulatory entities, that are organised around distinctive functions such as healthcare.

An IE approach aims to address three key questions: ‘what is happening’, ‘how is it happening’, and ‘why it is consequential’ [18]. To address these questions, it focuses on the *work* that people do. ‘Work’ refers to all activities that individuals or groups undertake within their particular institutional complex and involves skill, effort, time, knowledge, concerns and intent [21]. Another core tenet of IE is ‘standpoint’, which refers to one’s empirical social positioning in the ‘complex regime of institutions and governance’ [18: 2]. In our case, it refers to nurses’ location in the interprofessional/medical and organizational hierarchy. Exploring peoples’ work is the entry into, not the endpoint of IE; a key goal is investigating and explicating how institutional tasks and processes organise their work. This approach can reveal how nurses’ work of charting compliance was organised in such a way that it created an ethical peril.

### Data collection

The methods described here have been articulated in previous work (see Anon) using data from a larger study aimed at understanding the past implementation process and current practices related to the SSC. In part because IE takes a specific standpoint from which to understand how things are put together or are socially organised the way they are, it ‘is sampling an institutional process rather than a population…thus the goal is not representativeness of the broader population, but rather to understand a phenomenon or process in depth’[22: 4]. In this analysis we drew on a subset of these data, specifically interviews, open-ended survey responses, and observations. Data were collected between April 2017 and August 2019 and were anonymized. All names are pseudonyms.

The study received initial approval and subsequent renewals from C&C’s research ethics board (refs. 16–6140, April 4, 2017; 16–6140.2, March 5, 2019; 20–6102, April 7, 2021; and 20–6102.2, Jan 11, 2023). C&C is a multiple-site healthcare and research institution that employs some 25,000 staff, including scientists and clinicians who perform tens of thousands of medical procedures each year; each site focuses on different aspects of patient care and research. C&C is responsive to its regulatory, social, and financial environments and it is required to systematically report data on several quality and safety indicators to its governing/regulatory bodies (Anon).

Consent to participate in the interviews was written; consent for the observations and on-the-spot interviews was oral. The key ethical issue we encountered is the subject of this article. When a team member first learnt of the issues with nurses’ charting, it was immediately discussed with the whole team, and later with department colleagues and an expert in health professions research ethics. There were differing perspectives among the team during these discussions. Clinicians were not surprised by what we had found; the practice had, ostensibly, been normalized. Other team members struggled with the discovery and its broad implications. Our approach to reconciling this finding and our different perspectives was to tell a nuanced story of the moral hazard experienced by nurses as they knowingly contradicted professional duties. We thought it was important to understand and explain this practice more fully through further study.

We designed our data collection approach (and later, writeup) to minimise the likelihood that we would expose either the site of our research or the participants at the site by using pseudonyms, limiting identifiable information, and making a very large range of site visits across researchers. We opted to conduct on-the-spot interviews because these interviews could be done very quickly, over a couple of days, because we wanted to understand the nurses’ documentation practices in the moment.

#### Interviews

To understand the evolutionary process of the SSC, we conducted eight 30–60 minutes interviews with participants who were either involved in the design or the implementation of the SSC. These interviews documented their views on SSC compliance and its documentation. We revised the interview guide iteratively as our understanding of the research puzzle evolved. Interviews were recorded and transcribed verbatim.

#### Observations

Observations provide opportunities to see participants’ purposeful activities, how they do these, and to compare what say they do to what they actually do [23]. We observed and journaled fieldnotes of 51 surgical cases over a one-year period (185 hours). These were done by PhD (1) and Masters (2) trained ethnographers and a medical student and former pilot who had experience with aviation checklists. Observations included cardiac (12), plastic surgery (2), vascular (5), urology (2), gynecology (5), general (5), thoracic (13), and otolaryngology (7) specialties. Observations were focused on moments when surgical teams performed the SSC and when the nurses documented compliance. Observing multiple, whole cases facilitated a nuanced understanding of SSC adherence and the documentation process. Some participants were observed multiple times, others only once. These observations were complemented with hand-written and transcribed notes from informal, on-the-spot interviews with twelve surgical nurses.

#### Surveys

We used the data from open-ended questions from a survey. We adapted Singer et al’s. [24] survey and conducted two rounds of assessments of OR teams’ surgical safety beliefs/attitudes. Samples included nurses, anesthesiologists, anesthesia assistants, staff surgeons, OR attendants, perfusionists, and others. Round one (2017) included 98 participants (49 males, 46 females, 3 choices did not reflect gender). Round two (2019) included 133 participants (67 females, 62 males, 4 choices did not reflect gender). Respectively, response rates were 31% and 38%). Years of experience ranged from fewer than five to 25+ years.

### Data analysis

In line with IE, our inquiry started from the nurses’ standpoint to describe and track their work and purposeful activities to discern what is happening and to explicate how it came to be [20,25]. Our goal was to make visible the tensions and contradictions inherent in their experiences of documenting SSC compliance and to develop a critical explanation of how this was socially organised and put together systematically. Analysis involved an iterative process of indexing, mapping, and writing descriptive accounts [20]. Indexing involves reading the data and listing the nurses’ work processes that were related to documenting compliance. This strategy helped to keep nurses and their practices front of mind. It was also a way to start discerning tensions/conflicts and to identify and describe what ‘differently located people know and do’; e.g., surgeons [20], and to see how their practices may be linked.

We used mapping to identify how the nurses’ compliance charting processes were linked to other relevant texts and people; e.g., policies, EMR technologies, the surgical systems information specialists, the Ministry of Health and Long-term Care (MoHLTC), and to discern ‘when, where, and how they were activated’ [20: 6]. We crafted analytic pieces that further elaborated the indexes and maps that formed the basis for the account included in our findings. These analytic ‘bits’ were necessarily written with a view to linking back to the tensions/conflicts of documenting compliance. Questions that guided the analysis included: what are nurses doing; who else is involved and how are they linked into what the nurses do; what are nurses’ knowledge/training vis-a-vis charting; and how are they charting SSC compliance? What was the problematic generated from their practices, and what are its implications? Our purpose in this paper is not to blame, criticize or suggest nurses are deviant, rather it is per Rankin & Campbell [26] to explain the factors that influence nurses’ charting practices. The findings are reported in line with SRQR guidelines [27].

## Findings

### Nurses’ work of compliance documentation

In the next sections we track and explicate how management goals, mandated reporting requirements, EMR technology, interprofessional hierarchies and poor interprofessional communications influenced the nurses’ SSC compliance documentation work. The circulating nurses were responsible for documentation since it was the compliance form, not the SSC itself, that formed the basis for reporting. C&C’s coordination gave rise to a workaround that necessitated the potential violation of documentation standards and professional code of ethics. It also limited the nurses’ decision-making autonomy and obscured poor SSC adherence.

At C&C, SSC compliance is a reportable index that is linked to MoHLTC funding decisions. At the time of data collection, there was no auditing of reported data; C&C only needed to report the percentage of surgeries in which the SSC was performed. Fidelity to the SSC documentation policy and the College of Nursing of Ontario’s [8] (CNO) documentation standards require that the compliance form, which is housed in the EMR system, be completed only after the circulating nurse has observed the tasks being completed. Nonetheless, the nurses often pre-ensured compliance by checking all the boxes *ahead* of surgical procedures, before many components of the checklist would have been completed.

I ask Amelia about the checklist, and she toggles to the screen where she has to input the data. All the checkboxes are checked, even though the team hasn’t yet cut the skin and are still trying to put the patient to sleep. When I ask her about it, she tells me that the team “always” runs the checklist, and that she dislikes “to get disciplined” for not filling it properly… . She then continues: “We have a one hundred percent goal when it comes to accurate chart completion,” and so the nurses have to chart pre-emptively, as they navigate the patient record (Informal interview #4).

This excerpt suggests how the compliance documentation process involved conflating the completion of the compliance form with the completion of the SSC. Amelia’s claim that the team ‘always’ ran the SSC belied the fact that in our observations the debrief portion was rarely run. Indeed, there was consensus among the nurses that ‘we never do debriefs’ but when they showed us the interface, the ‘debrief’ boxes were always checked.

Pre-emptively charting showed how the nurses were oriented to C&C’s charting goals, efficiencies, and the personal (and attendant institutional) consequences if they were not met. If the boxes are not checked, according to Melissa, ‘you’ll have the manager come to you and ask why you didn’t do it’. Compliance charting ‘has to be done,’ Lisa explained, otherwise ‘it is a problem for the circulating nurse’. When charting was not done, it created extra work for nurses, in the form of ‘error reports’. Nurses who verified the charts were notified on the ‘verification’ screen and they would be asked to ‘fix it’ or ‘rectify the record’. In the Nursing notes section of the chart, they were compelled to ‘give a reason why it wasn’t charted’, said Helen.

Workflow processes, such as multiple handoffs during long/complex cases, the imperative to keep turning over ORs, and doubts about the relevancy of surgical debriefs not only oriented OR teams to a discourse that debriefs were too difficult under such dynamic conditions, but they also normalised skipping them. When debriefs were not skipped they were either done in professional silos, such as between surgeons and anesthesiologists and their respective fellows and residents, or they were simplified such that nurses were the only ones who participated because ‘lots of people have left the room’, including the surgeon, and it was often ‘chaotic to get everything wrapped up and sorted out before the next surgery’ (Deborah, Informal interview #12).

The misalignment of the EMR technology and workflow also oriented the nurses to pre-chart. The integration of the compliance form into the EMR was such that the circulating nurse in the OR at the end of a case needed to navigate backwards in a patient’s chart to confirm that the debrief was done or that the box was checked. Often this would be hours after the previous circulating nurse had confirmed the briefing and time out. This misalignment coupled with the ‘chaos’ at the end of cases, led to nurses being concerned that the debrief tickbox might be forgotten if left to later in the case. Similarly, the time lag between surgical cases and the chart reviews that would flag empty tick boxes further influenced pre-charting.

[You always check the SSC off as done] … otherwise you’ll get it back three weeks later as an error. Since by then you won’t remember whether it happened, you’ll always say ‘yes’. You’re not going to circle back to the surgeon and check that [they’ve] done it. They would look at you and say, ‘of course we did’. (Emily, Informal interview #9)

These charting errors, which were constructed as the nurses’ failure to complete the chart rather than the failure of OR teams to *‘*properly*’* run the SSC, may not be flagged for several weeks, at which time the nurses would be unlikely to recall the details of specific cases. And, since they would not circle back to the surgeons, pre-charting avoided potential inefficiency problems and interprofessional scuffles. In other words, the system obligated the nurses to attest to events they had not witnessed and led them to pre-chart.

One of the problems with the interface … is how if it is not checked off, then the alert goes to the nurse who finished the charting, and not necessarily to the person who was there when the event happened, when the actual step actually happened. So, you have to ‘correct’ something you were not in a position to see. The burden is on us. (Carole, Informal Interview #6)

‘In my opinion, this makes no sense; they force us to do this so that what is recorded in the chart does not reflect what happened but instead, the fact that we have to check this, we will do the check but regardless, it is not how we proceed with charting’ (Deborah, Informal Interview #12).

Because checklist performance was generally led by surgeons, assigning responsibility to nurses for attestation of completion was seen as misplaced by some: ‘If they didn’t do it [run the checklist ‘properly’ or at all], why is it up to us to bring it up… Why is it on us to do this?’ (Melissa, Informal Interview #11). Along with the nurses’ inability to enforce fidelity to the SSC, interprofessional hierarchies and poor interpersonal communications influenced their capacity to verify compliance. We did not observe a single case where the surgeons stopped to ensure that the nurses could keep track of progress on the SSC. Given the medical expertise required to fully understand surgical procedures and their intricacies, it was unclear how nurses could know whether something was omitted for good reason or because the surgeon or other team members had forgotten something.

Some nurses were disinclined to interactions that might be unpleasant because, as a perfusionist in our survey said, ‘sometimes the language directed at the nurses by the surgeons is a bit upsetting, they get frustrated and can be quite hostile/rude’. ‘The culture here allows for some quite disrespectful communication by specific individuals, with no apparent consequences’, said an anaesthetist. Another said, ‘surgeons are often the most disrespectful, however, it seems that little is ever done about it’. Nurses were also reluctant to ask or to question the surgeons because of their ‘aura of absolute authority, driven by a culture where some, not all…are above institutional rules, processes and procedures including those related to the checklists’ (Nurse, Attitudes Survey).

Amelia’s earlier comment showed that the nurses exercised some decision-making autonomy with respect to their workaround when they documented compliance opportunistically, while they were in the patient’s record charting other information. This was done in the name of efficiency and to avoid recall errors. The nurses could document compliance *whenever* they wanted but they could not document *whatever* they wanted. Compliance documentation was monitored by managers and/or surgical systems information specialists via the EMR system. The form, with ‘yes’ fields highlighted in red, denoted it was the singular option for the charting process to be completed. Nurses could check ‘no’ to report non-adherence with the SSC, but most did not perceive this as a viable option and, in some cases, they did not consider it an option at all.

…our charts cannot be completed without checking the box to indicate that it has been done. If any part of the team does not participate or if any part of the checklist is not completed, we are still obligated to indicate ’yes’ or the chart cannot be completed. (Nurse, Attitudes Survey)

Thus, compliance documentation, Betty observed, became ‘really only about having the boxes checked and not about what you have seen or heard’ (Informal Interview #5). The nurses knew nursing standards were being contravened, yet the system structures produced the practice of pre-charting as an acceptable workaround. Anna explained that C&C tried but could not discipline nurses who pre-charted or did not complete the SSC because the documentation system did not follow the CNO’s practice standards: ‘You can’t punish the nurses since the computer standards aren’t met’ (Informal Interview #8).

## Discussion

C&C’s social organisation of SSC compliance charting constitutes a moral hazard. Moral hazards, a concept which originated in the insurance and economics domains, characterise situations in which one engages in risky practices that will not harm the self but which may make other individuals incur their costs [28,29]. C&C’s system produced ethical concerns for nurses: it oriented them toward a workaround that technically violated the CNO’s documentation standards and professional code of ethics. Workarounds describe problem-solving strategies that deviate from established/expected practices; while they achieve efficiencies, they also represent lapses in standards of practice [30,31]. Nurses contravened documentation standards when they charted compliance before they witnessed completion of surgical cases, and when they attested to compliance in cases they had not witnessed.

This compliance workaround is influenced by multiple factors: SSC monitoring systems (the design of the compliance form within the EMR, provincial reporting mandate that allows only numerical reports, that are not audited), interprofessional and organisational hierarchies that undermine nurses’ authority and poor interprofessional communications. These local and extra-local texts, technologies, and relations paradoxically organised nurses’ documentation practices such that they arguably obscured SSC adherence and reported low-fidelity data. This social organisation forms part of the system that ‘builds a body of management information for submission’ to the MoHLTC. These imprecise data become the official account of what happens and form part of aggregate repository data for statistical analysis and comparison to other types of hospital performance; they ‘inform distant health officials regarding funding and hospital operations’[26: 12].

Bolton & Dewatripoint [32] suggest that the success of monitoring activities is shaped both by hierarchies and how monitoring is done. C&C’s compliance process responsibilises some of the least influential members of the OR team and obliges them to chart activities of team members over whom they have no authority. The surgeons, by virtue of their social position and the associated perception of their ‘absolute authority’ vis-à-vis nurses, are cushioned from monitoring their SSC practices. They fear no discipline when they elide adherence. Furthermore, nurses avoid consulting with surgeons to meet professional documentation standards because of sometimes unprofessional communications that engender poor relationships. Such relationships not only limit interprofessional collaborations they can also affect care and nursing retention [33].

In its current form, compliance documentation becomes not just a technical or administrative task, it draws nurses into the dominant practices of how C&C manages healthcare and patient safety. Rushton et al. [30: 372] write that there is widely held perception of nursing ‘as the most ethical and honest profession,’ yet their moral agency has long been recognized as constrained [34]. Although the charting workaround is an adaptive response to the possible inadequacy in the SSC administrative process, Rushton et al. [30] argue that workarounds are deviations from practice standards and their persistent use can result in broad detrimental effects, including insidious erosions of individual and disciplinary integrity. They also put nurses at risk because falsifying patient records constitutes professional misconduct [8]. We offer some possible solutions for how to address some of the issues and concerns of SSC administration (Table 1).

**Table 1:** Summary of problems and solutions.

Issue	Proposed solution
Too many items on checklist.	Review checklist for items that are critical to include. This includes items that are likely to be missed, or rare but critical items that if missed, would lead to significant errors in care.
Checklist items are unclear.	Review checklist to ensure that directions are clear, focused, and actionable. One action per checklist item only should exist.
Checklist contains both reminder items and communication prompts.	Determine whether the checklist is meant to act as a memory supplement or communication prompt and design the content accordingly.
Pre-charting of checklist compliance.	Build documentation flow of checklist compliance into EMR system appropriately (i.e., each stage of the checklist should not be documented on the same page in the patient’s chart, as stages often occur several hours apart). Improve communication between team members and timing of checklist to be performed. Remove requirement for checklist use to be charted. Remove mandated reporting of compliance with the checklist.

## Strengths and limitations

We used IE to understand how a group of OR nurses’ work of documenting SSC compliance was socially organised such that the process came to be a moral hazard. We used their accounts of their activities to trace the connections and disconnections between broader institutional forces (e.g., policies) and their compliance charting practices. We linked this ostensible violation of nursing charting principles to provincial reporting mandates, EMR technology, as well as interprofessional hierarchies and communication. This breach is illustrative of the focus on reporting compliance rather than SSC adherence and puts nurses in an ethically conflictual position.

IE, McCoy writes in Rankin & Campbell [26: 39] ‘is a determinedly empirical project’. Its ‘interest begins and remains in the actual activities and workings of people and institutions that construct the social’ world. We used several strategies that aligned with institutional ethnography to ensure rigour. Analytic rigour, a strength of our study, is demonstrated in our detailed, transparent descriptions of what nurses were actually doing and our explication of the institutional processes/forces that organised their activities in this way. Although being a single site study might be perceived as a limitation, we took various methodological steps to strengthen the quality of our study. We paid particular attention to data credibility and sampling [35,36]. Data sources were multiple, there was prolonged engagement in the study site and transcripts were audited for accuracy in transcription. Researcher and data triangulation were used to provide depth, breadth, and nuanced understanding of the SSC processes. This study could be reproduced as a comparative study to evaluate whether other factors play into documentation practices, or whether different EMRs are better at preventing charting workarounds.

IE does not make direct links between objectivity and validity of findings; the notion of ‘bias’ is qualitative inquiry is conceived not as problematic but as a resource to inform analytic insights and reflexivity [37]. The aim of IE is to produce faithful accounts of how things really are and relies on authenticity and reflexivity processes to achieve this [22]. Another strength of our study was its multi-disciplinary research team that comprised surgeon/scientists, nurse/scientists, (health) sociologists, anthropologists, and medical students. The composition of our team and the nature of participation allowed us to learn different perspectives, look at the data through different disciplinary lenses, and challenge our assumptions about what we were seeing. Different team members conducted the interviews, which provided different sorts of data, including sometimes very frank discussions depending on who the interviewer-interviewee were. Different team members also conducted the observations and sometimes there were two members simultaneously observing a case.

## Conclusion

In this article we showed how one group of nurses’ work of documenting SSC compliance was socially organised in ways that met their hospital’s accuracy in charting goals and legislative reporting needs while putting them in ethically untenable positions. This represents a moral hazard whereby OR teams did not fully adhere to fidelity of SSC completion and the circulating nurses bore the ethical risks associated with transgressing their professional practice standards and code of ethics to meet these reporting goals. Nurses’ autonomy and moral agency were constrained which Rushton et al. [30] and Peter & Liaschenko [34] argue can contribute to job dissatisfaction and unhealthy work environments. This coordination of compliance likely functioned to obscure poor checklist adherence by eclipsing actual SSC performance in favour of recording compliance.

In qualitative inquiry issues of generalisability are understood in terms of analytic or theoretical relevance, where research contributes to conceptual knowledge or novel or existing theory; or in terms of naturalistic generalisability, where findings or concepts are transferable or may have relevance to other situations [38–40]. Our finding of compliance documentation as a moral hazard has analytic and naturalistic applicability to other situations where workers are tasked with textually demonstrating that they have complied with legislated safety protocols. Finally, the study contributes empirically to SSC knowledge and to the ongoing conversations about the problem of nursing workarounds, interprofessional communication, and institutional and structural power dynamics [see e.g., 41]. These IE derived findings suggest future research focus not on how SSC compliance can be better documented but on how the SSC process can be differently organised. Further, the findings reflect only the experiences of OR nurses; future research could attend to the SSC-related activities of other OR team members to understand more fully what is actually happening, how their work is linked into that of nurses, and how and whether it is organised by the same institutional dynamics.

## Supporting information

S1 File Global inclusivity form. (DOCX)
